# Supplementary material for: Genetic Characterization and Pathogenesis of H5N1 High Pathogenicity Avian Influenza Virus Isolated in South Korea during 2021–2022
Source: Viruses. 2023 Jun 20;15(6):1403. doi: 10.3390/v15061403 (PMC10304347; doi:10.3390/v15061403)
Supplement: Supplementary file 1 [file viruses-15-01403-s001.zip › Supplemantary Figures and tables_230526/Figure S1.pdf]

(A)

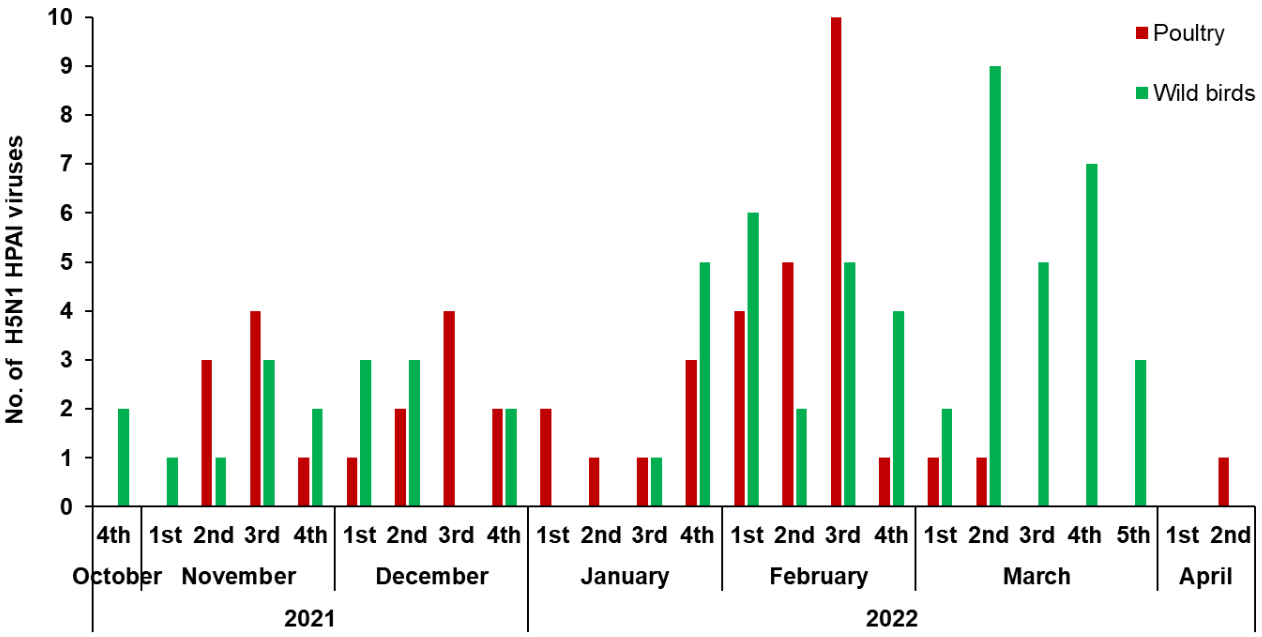

(B)

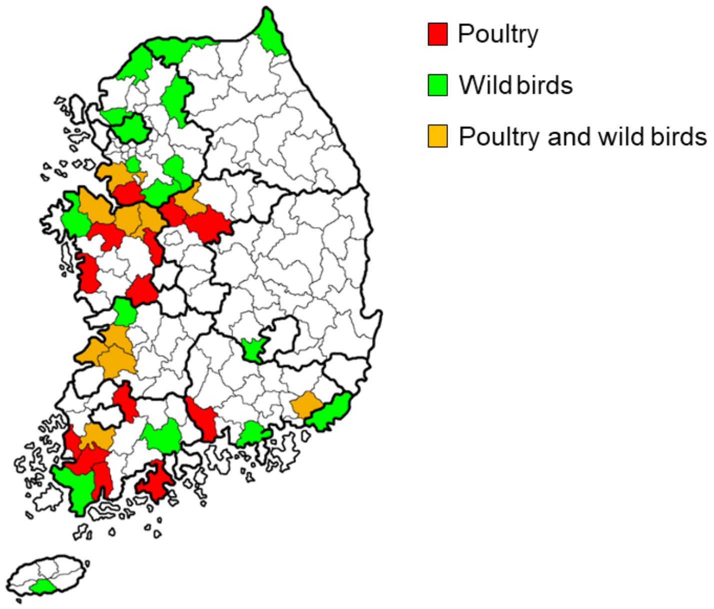

Figure S1. H5N1 high pathogenicity avian influenza viruses isolated from wild birds and poultry in Korea during the 2021–2022 winter season by date (**A**) and by region (**B**). Each bar represents the number of H5N1 HPAI viruses isolated each week (red, poultry; green, wild birds) (**A**). Geographic depiction of the region in Korea where H5N1 HPAI viruses were detected in poultry (red), wild birds (green), or both poultry and wild birds (yellow) (**B**). Data were obtained from the Korean Animal Health Integrated System (KAHIS) and Ministry of Agriculture, Food and Rural Affairs of Korea webpage (www.mafra.go.kr/FMD-AI2).
