# Supplementary material for: Genetic Characterization and Pathogenesis of H5N1 High Pathogenicity Avian Influenza Virus Isolated in South Korea during 2021–2022
Source: Viruses. 2023 Jun 20;15(6):1403. doi: 10.3390/v15061403 (PMC10304347; doi:10.3390/v15061403)
Supplement: Supplementary file 1 [file viruses-15-01403-s001.zip › Supplemantary Figures and tables_230526/Table S1.pdf]

**Table S1**. Clade 2.3.4.4b H5N1 highly pathogenic avian influenza viruses isolated in this study

| Host | | Virus name | Collection date | Region | Genetic group | Sample type | latitude | longitude | Cleavage site | GISAID PB2 accession | GISAID PB1 accession | GISAID PA accession | GISAID HA accession | GISAID NP accession | GISAID NA accession | GISAID MP accession | GISAID NS accession |
|---|---|---|---|---|---|---|---|---|---|---|---|---|---|---|---|---|---|
| Wild bird | Mandarin Duck | A/mandarin duck/Korea/WA585/2021 | 2021-10-26 | CN | I | Swab | 36°44′56″N | 127°7′37″E | PLRERRRKR*GLF | EPI2205104 | EPI2205103 | EPI2205102 | EPI2205101 | EPI2205100 | EPI2205099 | EPI2205098 | EPI2205097 |
| | Mandarin Duck | A/mandarin duck/Korea/H519/2021 | 2021-11-05 | JB | I | Feces | 35°37′26″N | 126°51′40″E | PLRERRRKR*GLF | EPI2540373 | EPI2540374 | EPI2540372 | EPI2540376 | EPI2540369 | EPI2540375 | EPI2540371 | EPI2540370 |
| | Mandarin Duck | A/mandarin duck/Korea/WA622/2021 | 2021-11-09 | JB | I | Swab | 35°40′38″N | 126°53′30″E | PLRERRRKR*GLF | EPI2540381 | EPI2540382 | EPI2540380 | EPI2540384 | EPI2540377 | EPI2540383 | EPI2540379 | EPI2540378 |
| | Mandarin Duck | A/mandarin duck/Korea/H552/2021 | 2021-11-15 | GG | I | Feces | 37°13′49″N | 127°26′28″E | PLRERRRKR*GLF | EPI2540389 | EPI2540390 | EPI2540388 | EPI2540392 | EPI2540385 | EPI2540391 | EPI2540387 | EPI2540386 |
| | Mallard | A/mallard/Korea/WA44/2022 | 2022-01-13 | CN | II | Swab | 36°48′32″N | 126°58′25″E | PLRERRRKR*GLF | EPI2540397 | EPI2540398 | EPI2540396 | EPI2540400 | EPI2540393 | EPI2540399 | EPI2540395 | EPI2540394 |
| | Wild duck | A/wild duck/Korea/H152/2022 | 2022-02-08 | GG | II | Feces | 37°12′53″N | 127°1′10″E | PLRERRRKR*GLF | EPI2540405 | EPI2540406 | EPI2540404 | EPI2540408 | EPI2540401 | EPI2540407 | EPI2540403 | EPI2540402 |
| Poultry | Quail | A/quail/Korea/H526/2021 | 2021-11-09 | CB | I | Organ | 36°59′28″N | 127°32′41″E | PLRERRRKR*GLF | EPI1938269 | EPI1938270 | EPI1938271 | EPI1938272 | EPI1938273 | EPI1938274 | EPI1938275 | EPI1938276 |
| | Duck | A/duck/Korea/H535-2/2021 | 2021-11-10 | CB | I | Organ | 36°59′7″N | 127°32′8″E | PLRERRRKR*GLF | EPI2540413 | EPI2540414 | EPI2540412 | EPI2540416 | EPI2540409 | EPI2540415 | EPI2540411 | EPI2540410 |
| | Duck | A/duck/Korea/H541-3/2021 | 2021-11-11 | JN | I | Organ | 34°54′39″N | 126°43′4″E | PLRERRRKR*GLF | EPI2540421 | EPI2540422 | EPI2540420 | EPI2540424 | EPI2540417 | EPI2540423 | EPI2540419 | EPI2540418 |
| | Duck | A/duck/Korea/H543/2021 | 2021-11-14 | CB | I | Organ | 37°0′7″N | 127°34′15″E | PLRERRRKR*GLF | EPI2540781 | EPI2540782 | EPI2540780 | EPI2540784 | EPI2540777 | EPI2540783 | EPI2540779 | EPI2540778 |
| | Duck | A/duck/Korea/H550/2021 | 2021-11-16 | JN | I | Organ | 34°29′58″N | 126°41′38″E | PLRERRRKR*GLF | EPI2540429 | EPI2540430 | EPI2540428 | EPI2540432 | EPI2540425 | EPI2540431 | EPI2540427 | EPI2540426 |
| | Duck | A/duck/Korea/H554/2021 | 2021-11-17 | JN | I | Organ | 34°55′23″N | 126°43′1″E | PLRERRRKR*GLF | EPI2540437 | EPI2540438 | EPI2540436 | EPI2540440 | EPI2540433 | EPI2540439 | EPI2540435 | EPI2540434 |
| | Chicken | A/chicken/Korea/H559/2021 | 2021-11-21 | CB | I | Organ | 36°59′29″N | 127°33′46″E | PLRERRRKR*GLF | EPI2540445 | EPI2540446 | EPI2540444 | EPI2540448 | EPI2540441 | EPI2540447 | EPI2540443 | EPI2540442 |
| | Duck | A/duck/Korea/H560/2021 | 2021-11-22 | JN | I | Organ | 35°14′40″N | 127°0′47″E | PLRERRRKR*GLF | EPI2540453 | EPI2540454 | EPI2540452 | EPI2540456 | EPI2540449 | EPI2540455 | EPI2540451 | EPI2540450 |
| | Chicken | A/chicken/Korea/H589/2021 | 2021-12-04 | CN | I | Organ | 36°45′5″N | 127°6′53″E | PLRERRRKR*GLF | EPI2540461 | EPI2540462 | EPI2540460 | EPI2540464 | EPI2540457 | EPI2540463 | EPI2540459 | EPI2540458 |
| | Chicken | A/chicken/Korea/H590/2021 | 2021-12-05 | JN | I | Organ | 34°44′35″N | 126°27′42″E | PLRERRRKR*GLF | EPI2540469 | EPI2540470 | EPI2540468 | EPI2540472 | EPI2540465 | EPI2540471 | EPI2540467 | EPI2540466 |
| | Chicken | A/chicken/Korea/H605/2021 | 2021-12-11 | CN | I | Organ | 36°43′41″N | 127°8′49″E | PLRERRRKR*GLF | EPI2540477 | EPI2540478 | EPI2540476 | EPI2540480 | EPI2540473 | EPI2540479 | EPI2540475 | EPI2540474 |
| | Duck | A/duck/Korea/H608/2021 | 2021-12-13 | JN | III | Organ | 34°52′6″N | 126°32′4″E | PLRERRRKR*GLF | EPI2540485 | EPI2540486 | EPI2540484 | EPI2540488 | EPI2540481 | EPI2540487 | EPI2540483 | EPI2540482 |
| | Duck | A/duck/Korea/H609/2021 | 2021-12-13 | JN | III | Organ | 34°52′9″N | 126°32′6″E | PLRERRRKR*GLF | EPI2540493 | EPI2540494 | EPI2540492 | EPI2540496 | EPI2540489 | EPI2540495 | EPI2540491 | EPI2540490 |
| | Chicken | A/chicken/Korea/H617/2021 | 2021-12-14 | CN | I | Organ | 36°51′28″N | 126°59′51″E | PLRGRRRKR*GLF | EPI2540501 | EPI2540502 | EPI2540500 | EPI2540504 | EPI2540497 | EPI2540503 | EPI2540499 | EPI2540498 |
| | Duck | A/duck/Korea/H621/2021 | 2021-12-15 | JN | I | Organ | 34°54′5″N | 126°41′9″E | PLRERRRKR*GLF | EPI2540509 | EPI2540510 | EPI2540508 | EPI2540512 | EPI2540505 | EPI2540511 | EPI2540507 | EPI2540506 |
| | Chicken | A/chicken/Korea/H623/2021 | 2021-12-16 | SJ | I | Organ | 36°33′29″N | 127°18′33″E | PLRERRRKR*GLF | EPI2540517 | EPI2540518 | EPI2540516 | EPI2540520 | EPI2540513 | EPI2540519 | EPI2540515 | EPI2540514 |
| | Quail | A/quail/Korea/H624/2021 | 2021-12-17 | GG | I | Organ | 37°48′30″N | 126°53′45″E | PLRERRRKR*GLF | EPI2540525 | EPI2540526 | EPI2540524 | EPI2540528 | EPI2540521 | EPI2540527 | EPI2540523 | EPI2540522 |
| | Chicken | A/chicken/Korea/H625/2021 | 2021-12-20 | JN | III | Organ | 34°50′8″N | 126°34′34″E | PLRERRRKR*GLF | EPI2540533 | EPI2540534 | EPI2540532 | EPI2540536 | EPI2540529 | EPI2540535 | EPI2540531 | EPI2540530 |
| | Chicken | A/chicken/Korea/H638/2021 | 2021-12-23 | SJ | I | Organ | 36°30′44″N | 127°22′35″E | PLRERRRKR*GLF | EPI2540541 | EPI2540542 | EPI2540540 | EPI2540544 | EPI2540537 | EPI2540543 | EPI2540539 | EPI2540538 |
| | Duck | A/duck/Korea/H640/2021 | 2021-12-27 | JB | I | Organ | 35°35′24″N | 126°35′22″E | PLRERRRKR*GLF | EPI2540549 | EPI2540550 | EPI2540548 | EPI2540552 | EPI2540545 | EPI2540551 | EPI2540547 | EPI2540546 |
| | Duck | A/duck/Korea/H649/2021 | 2021-12-29 | JN | II | Organ | 34°49′53″N | 126°29′59″E | PLRERRRKR*GLF | EPI2540557 | EPI2540558 | EPI2540556 | EPI2540560 | EPI2540553 | EPI2540559 | EPI2540555 | EPI2540554 |
| | Duck | A/duck/Korea/H13/2022 | 2022-01-06 | JB | II | Organ | 35°40′26″N | 126°54′2″E | PLRERRRKR*GLF | EPI2540565 | EPI2540566 | EPI2540564 | EPI2540568 | EPI2540561 | EPI2540567 | EPI2540563 | EPI2540562 |

| | | | | | | | | | | | | | | | | |
|---|---|---|---|---|---|---|---|---|---|---|---|---|---|---|---|---|
| Duck | A/duck/Korea/H38/2022 | 2022-01-14 | JN | I | Organ | 34°47′33″N | 126°36′11″E | PLRERRRKR*GLF | EPI2540573 | EPI2540574 | EPI2540572 | EPI2540576 | EPI2540569 | EPI2540575 | EPI2540571 | EPI2540570 |
| Duck | A/duck/Korea/H56/2022 | 2022-01-21 | CB | II | Organ | 36°57′8″N | 127°26′33″E | PLRERRRKR*GLF | EPI2540581 | EPI2540582 | EPI2540580 | EPI2540584 | EPI2540577 | EPI2540583 | EPI2540579 | EPI2540578 |
| Chicken | A/chicken/Korea/H57/2022 | 2022-01-21 | GG | II | Organ | 37°5′11″N | 126°52′51″E | PLRERRRKR*GLF | EPI2540589 | EPI2540590 | EPI2540588 | EPI2540592 | EPI2540585 | EPI2540591 | EPI2540587 | EPI2540586 |
| Chicken | A/chicken/Korea/H58/2022 | 2022-01-21 | GG | II | Organ | 37°10′20″N | 126°51′16″E | PLRERRRKR*GLF | EPI2540597 | EPI2540598 | EPI2540596 | EPI2540600 | EPI2540593 | EPI2540599 | EPI2540595 | EPI2540594 |
| Chicken | A/chicken/Korea/H63/2022 | 2022-01-24 | CN | II | Organ | 36°43′47″N | 127°6′59″E | PLRERRRKR*GLF | EPI2540605 | EPI2540606 | EPI2540604 | EPI2540608 | EPI2540601 | EPI2540607 | EPI2540603 | EPI2540602 |
| Duck | A/duck/Korea/H79/2022 | 2022-01-25 | JB | III | Organ | 35°41′47″N | 126°43′0″E | PLRERRRKR*GLF | EPI2540613 | EPI2540614 | EPI2540612 | EPI2540616 | EPI2540609 | EPI2540615 | EPI2540611 | EPI2540610 |
| Duck | A/duck/Korea/H87/2022 | 2022-01-29 | JB | II | Organ | 35°45′25″N | 126°59′21″E | PLRERRRKR*GLF | EPI2540621 | EPI2540622 | EPI2540620 | EPI2540624 | EPI2540617 | EPI2540623 | EPI2540619 | EPI2540618 |
| Chicken | A/chicken/Korea/H88/2022 | 2022-01-30 | CN | II | Organ | 36°45′32″N | 126°50′11″E | PLRERRRKR*GLF | EPI2540629 | EPI2540630 | EPI2540628 | EPI2540632 | EPI2540625 | EPI2540631 | EPI2540627 | EPI2540626 |
| Duck | A/duck/Korea/H90/2022 | 2022-01-31 | CB | II | Organ | 36°54′44″N | 127°27′4″E | PLRERRRKR*GLF | EPI2540637 | EPI2540638 | EPI2540636 | EPI2540640 | EPI2540633 | EPI2540639 | EPI2540635 | EPI2540634 |
| Chicken | A/chicken/Korea/H91/2022 | 2022-02-02 | CN | II | Organ | 36°57′46″N | 127°6′38″E | PLRERRRKR*GLF | EPI2540645 | EPI2540646 | EPI2540644 | EPI2540648 | EPI2540641 | EPI2540647 | EPI2540643 | EPI2540642 |
| Korean native chicken | A/Korean native chicken/Korea/H97/2022 | 2022-02-03 | CN | II | Organ | 36°43′52″N | 127°8′14″E | PLREKRRKR*GLF | EPI2540653 | EPI2540654 | EPI2540652 | EPI2540656 | EPI2540649 | EPI2540655 | EPI2540651 | EPI2540650 |
| Chicken | A/chicken/Korea/H124/2022 | 2022-02-04 | JB | III | Organ | 35°41′24″N | 126°43′8″E | PLRGKRRKR*GLF | EPI2540661 | EPI2540662 | EPI2540660 | EPI2540664 | EPI2540657 | EPI2540663 | EPI2540659 | EPI2540658 |
| Duck | A/duck/Korea/H125/2022 | 2022-02-05 | JB | IV | Organ | 35°39′30″N | 126°45′58″E | PLRERRRKR*GLF | EPI2205112 | EPI2205111 | EPI2205110 | EPI2205109 | EPI2205108 | EPI2205107 | EPI2205106 | EPI2205105 |
| Korean native chicken | A/Korean native chicken/Korea/H127/2022 | 2022-02-06 | CN | IV | Organ | 36°27′28″N | 126°34′1″E | PLRERRRKR*GLF | EPI2540669 | EPI2540670 | EPI2540668 | EPI2540672 | EPI2540665 | EPI2540671 | EPI2540667 | EPI2540666 |
| Duck | A/duck/Korea/H128/2022 | 2022-02-07 | CB | II | Organ | 36°53′52″N | 127°28′6″E | PLREKRRKR*GLF | EPI2540677 | EPI2540678 | EPI2540676 | EPI2540680 | EPI2540673 | EPI2540679 | EPI2540675 | EPI2540674 |
| Chicken | A/chicken/Korea/H129/2022 | 2022-02-07 | GG | II | Organ | 37°6′26″N | 127°0′38″E | PLRERRRKR*GLF | EPI2540685 | EPI2540686 | EPI2540684 | EPI2540688 | EPI2540681 | EPI2540687 | EPI2540683 | EPI2540682 |
| Chicken | A/chicken/Korea/H130/2022 | 2022-02-07 | JB | IV | Organ | 35°34′5″N | 126°45′55″E | PLRERRRKR*GLF | EPI2540693 | EPI2540694 | EPI2540692 | EPI2540696 | EPI2540689 | EPI2540695 | EPI2540691 | EPI2540690 |
| Duck | A/duck/Korea/H138/2022 | 2022-02-08 | CB | II | Organ | 36°46′40″N | 127°24′45″E | PLRERRRKR*GLF | EPI2540701 | EPI2540702 | EPI2540700 | EPI2540704 | EPI2540697 | EPI2540703 | EPI2540699 | EPI2540698 |
| Quail | A/quail/Korea/H139/2022 | 2022-02-08 | CN | II | Organ | 36°45′52″N | 126°45′4″E | PLRERRRKR*GLF | EPI2540709 | EPI2540710 | EPI2540708 | EPI2540712 | EPI2540705 | EPI2540711 | EPI2540707 | EPI2540706 |
| Chicken | A/chicken/Korea/H141/2022 | 2022-02-09 | CN | II | Organ | 36°47′19″N | 126°55′54″E | PLRERRRKR*GLF | EPI2540717 | EPI2540718 | EPI2540716 | EPI2540720 | EPI2540713 | EPI2540719 | EPI2540715 | EPI2540714 |
| Duck | A/duck/Korea/H153/2022 | 2022-02-12 | CB | II | Organ | 36°52′29″N | 127°54′18″E | PLRERRRKR*GLF | EPI2540725 | EPI2540726 | EPI2540724 | EPI2540728 | EPI2540721 | EPI2540727 | EPI2540723 | EPI2540722 |
| Chicken | A/chicken/Korea/H154-1/2022 | 2022-02-13 | CN | II | Organ | 36°46′28″N | 126°45′6″E | PLREKRRKR*GLF | EPI2540733 | EPI2540734 | EPI2540732 | EPI2540736 | EPI2540729 | EPI2540735 | EPI2540731 | EPI2540730 |
| Chicken | A/chicken/Korea/H155/2022 | 2022-02-12 | CB | II | Organ | 36°53′31″N | 127°28′6″E | PLRERRRKR*GLF | EPI2540741 | EPI2540742 | EPI2540740 | EPI2540744 | EPI2540737 | EPI2540743 | EPI2540739 | EPI2540738 |
| Duck | A/duck/Korea/H196/2022 | 2022-02-18 | CN | IV | Organ | 36°5′53″N | 127°5′55″E | PLRGKRRKR*GLF | EPI2540749 | EPI2540750 | EPI2540748 | EPI2540752 | EPI2540745 | EPI2540751 | EPI2540747 | EPI2540746 |
| Duck | A/duck/Korea/H200/2022 | 2022-02-21 | GN | II | Organ | 35°3′26″N | 127°55′1″E | PLRERRRKR*GLF | EPI2540757 | EPI2540758 | EPI2540756 | EPI2540760 | EPI2540753 | EPI2540759 | EPI2540755 | EPI2540754 |
| Duck | A/duck/Korea/H232/2022 | 2022-03-02 | JN | IV | Organ | 34°44′4″N | 127°21′3″E | PLRERRRKR*GLF | EPI2540765 | EPI2540766 | EPI2540764 | EPI2540768 | EPI2540761 | EPI2540767 | EPI2540763 | EPI2540762 |
| Chicken | A/chicken/Korea/H384/2022 | 2022-04-07 | GN | II | Organ | 35°17′5″N | 128°49′50″E | PLRERRRKR*GLF | EPI2540773 | EPI2540774 | EPI2540772 | EPI2540776 | EPI2540769 | EPI2540775 | EPI2540771 | EPI2540770 |

JB, Jeonbuk; JN, Jeonnam; GG, Gyeonggi; CB, Chungbuk; CN, Chungnam; SJ, Sejong
