# Supplementary material for: Genetic Characterization and Pathogenesis of H5N1 High Pathogenicity Avian Influenza Virus Isolated in South Korea during 2021–2022
Source: Viruses. 2023 Jun 20;15(6):1403. doi: 10.3390/v15061403 (PMC10304347; doi:10.3390/v15061403)
Supplement: Supplementary file 1 [file viruses-15-01403-s001.zip › Supplemantary Figures and tables_230526/Figure S2.pdf]

(A) PB2

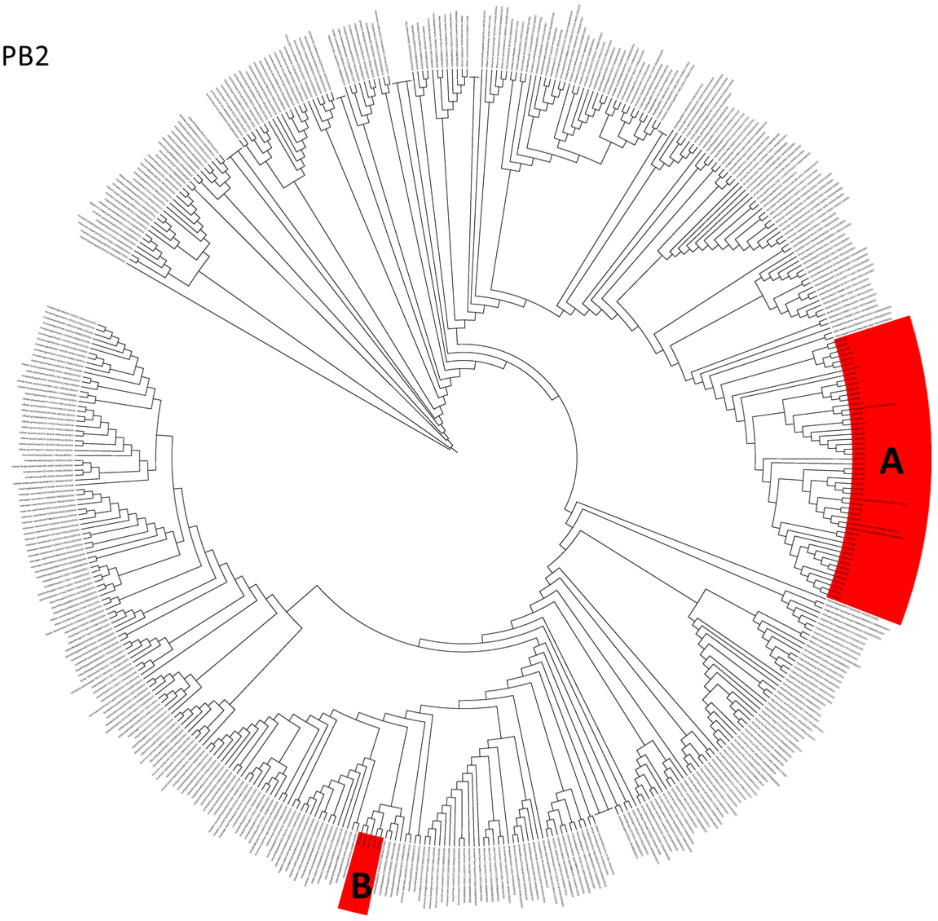

(B) PB1

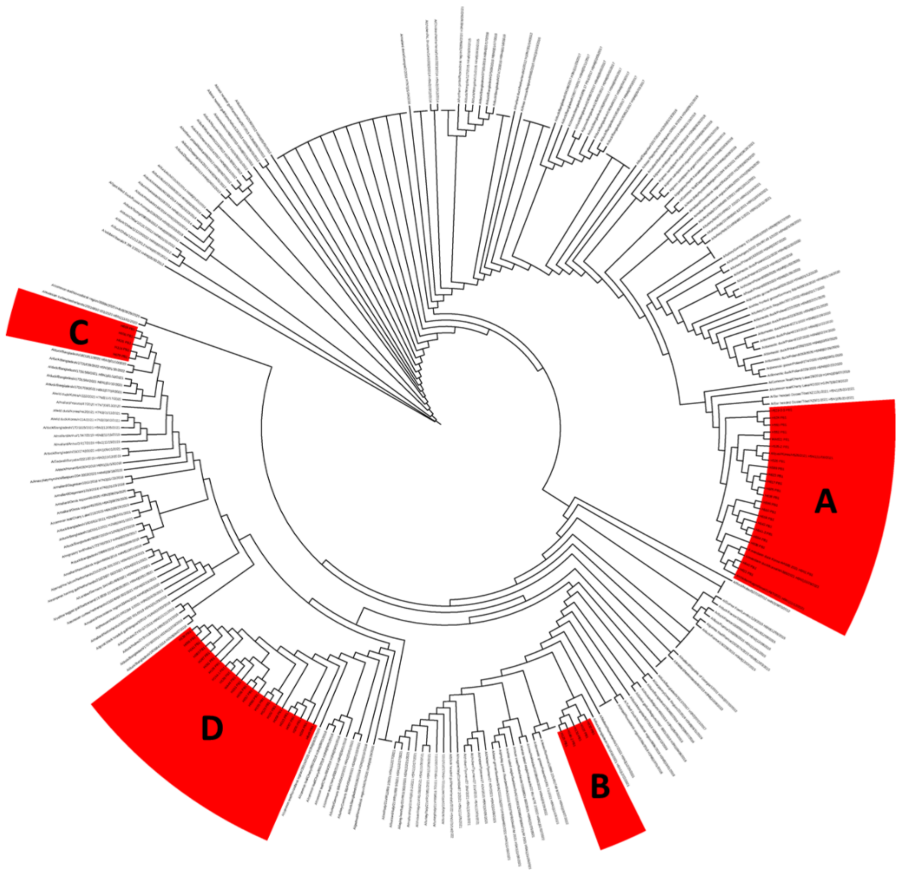

(C) PA

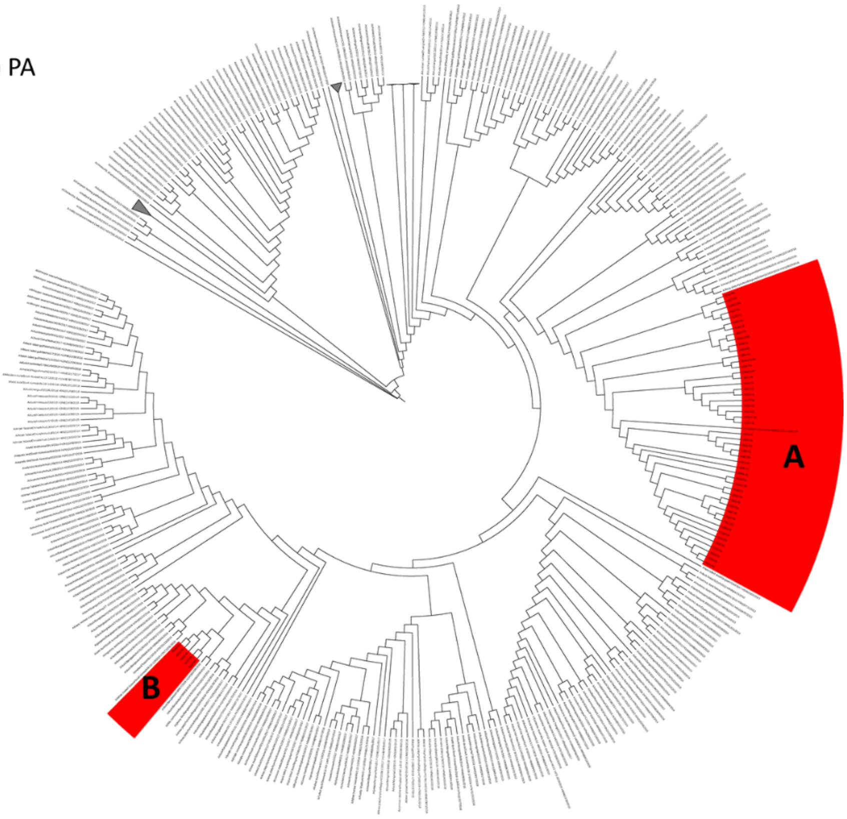

(D) NP

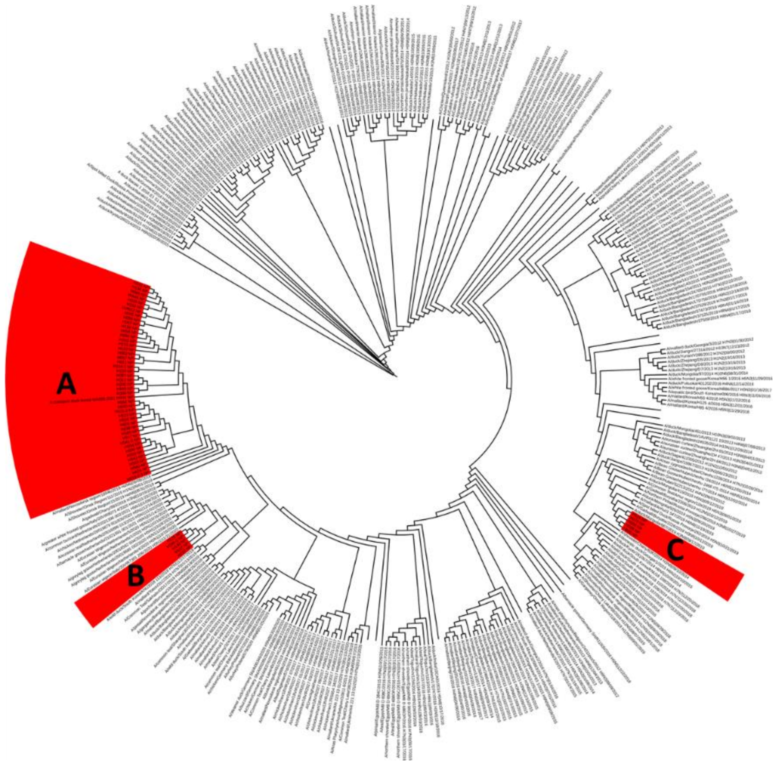

(E) MP

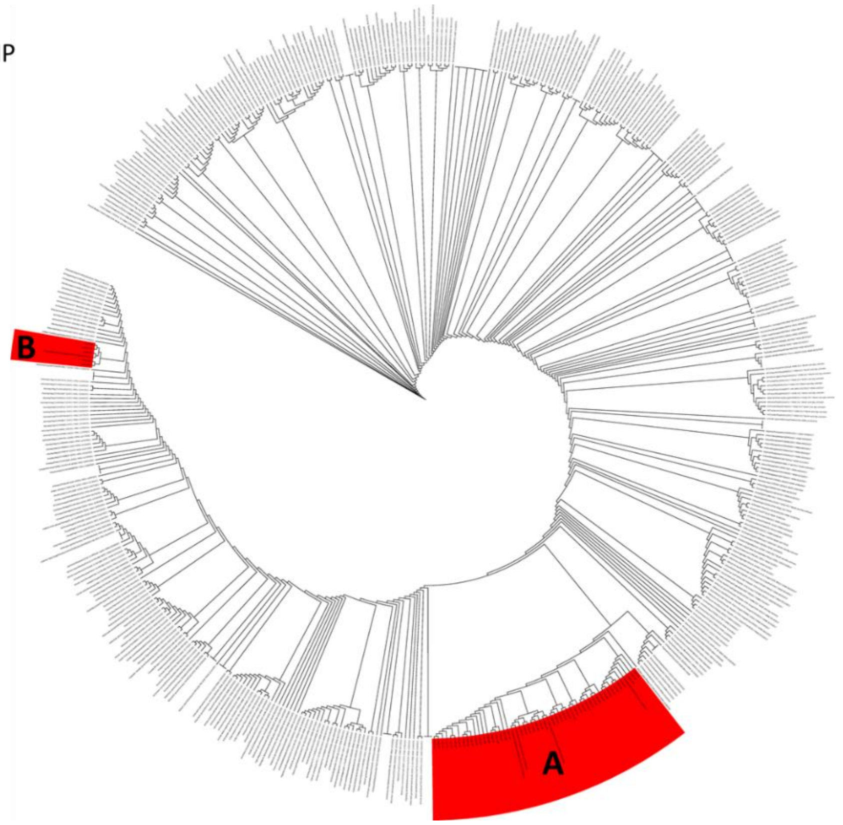

(F) NS

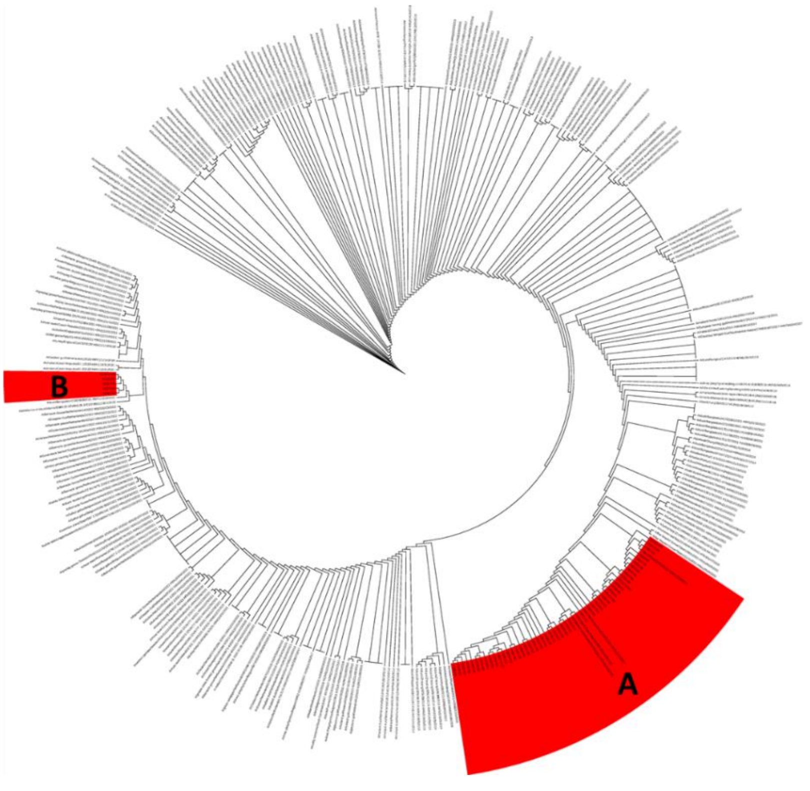

Figure S2. Maximum likelihood (ML) phylogenetic tree of PB2 (**A**), PB1 (**B**), PA (**C**), NP (**D**), MP (**E**), and NS (**F**) genes.
